# Effects of Orientation and Anisometry of Magnetic Resonance Imaging Acquisitions on Diffusion Tensor Imaging and Structural Connectomes

**DOI:** 10.1371/journal.pone.0170703

**Published:** 2017-01-24

**Authors:** Raúl Tudela, Emma Muñoz-Moreno, Xavier López-Gil, Guadalupe Soria

**Affiliations:** 1 CIBER de Bioingeniería, Biomateriales y Nanomedicina (CIBER-BBN), Barcelona, Spain; 2 Experimental MRI 7T Unit, IDIBAPS, Barcelona, Spain; University of North Carolina at Chapel Hill, UNITED STATES

## Abstract

Diffusion-weighted imaging (DWI) quantifies water molecule diffusion within tissues and is becoming an increasingly used technique. However, it is very challenging as correct quantification depends on many different factors, ranging from acquisition parameters to a long pipeline of image processing. In this work, we investigated the influence of voxel geometry on diffusion analysis, comparing different acquisition orientations as well as isometric and anisometric voxels. Diffusion-weighted images of one rat brain were acquired with four different voxel geometries (one isometric and three anisometric in different directions) and three different encoding orientations (coronal, axial and sagittal). Diffusion tensor scalar measurements, tractography and the brain structural connectome were analyzed for each of the 12 acquisitions. The acquisition direction with respect to the main magnetic field orientation affected the diffusion results. When the acquisition slice-encoding direction was not aligned with the main magnetic field, there were more artifacts and a lower signal-to-noise ratio that led to less anisotropic tensors (lower fractional anisotropic values), producing poorer quality results. The use of anisometric voxels generated statistically significant differences in the values of diffusion metrics in specific regions. It also elicited differences in tract reconstruction and in different graph metric values describing the brain networks. Our results highlight the importance of taking into account the geometric aspects of acquisitions, especially when comparing diffusion data acquired using different geometries.

## Introduction

Diffusion-weighted imaging (DWI) quantifies the diffusion of water molecules within tissues. As this diffusion is directionally constrained by cellular membranes and other structures, different properties of the brain microstructure can be studied by DWI [1–4]. For instance, the main neuronal fiber tracts can be reconstructed [5, 6], since diffusion in brain white matter occurs mainly in the direction parallel to the axons. To this end, different methods have been proposed for DWI analysis, including quantification of scalar parameters calculated from the diffusion tensor model, tractography, as well as connectomics that evaluate the network of connections in the brain [7, 8]. Indeed, DWI-based connectomics have been widely used in recent years to study the connection among different regions of the brain and their alterations in pathologies [9–12].

DWI is becoming an increasingly used technique. However, it is very challenging as the quantification and analysis results depend on both acquisition and processing parameters. Typical processing steps include preprocessing (i.e. adapting the file format) and quality control (i.e. identification of outliers, signal dropouts, subtle system drifts and missing slices), distortion and motion correction, segmentation, diffusion tensor estimation, calculation of scalar indices, tractography, connectome extraction and integration in multimodal studies [4]. For this reason, it is of great interest to quantify and evaluate the effect of these different parameters on DWI results.

From the acquisition point of view, DWI is very demanding in terms of magnetic resonance imaging (MRI) systems, especially for *in vivo* applications that require high spatial resolution within short acquisition times and strong gradient powers in multiple directions [13]. This makes the diffusion datasets susceptible to artifacts and low signal–to-noise ratios (SNR), many of which are affected by the pulse sequence and the acquisition method. The most common acquisition method is echo planar imaging (EPI), which enables the acquisition of diffusion-weighted information that is sufficiently rapid to avoid significant movement artifacts. However, the fast readout of k-space in EPI sequences produces a low bandwidth in the phase-encoding direction, making the images more sensitive to off-resonance, susceptibility and eddy current effects [14, 15]. These effects can partly be overcome by using navigator techniques in the sequence, which increases the acquisition time.

The different factors affecting acquisition include the number of repetitions, the number of diffusion gradient directions, strength, the number of b-values and the voxel size used.

- The number of repetitions is directly related to the SNR; the more scan repetitions, the higher the SNR, producing more reliable diffusion data and tractography [16, 17].
- The effect of diffusion gradient number on diffusion anisotropic metrics, estimation of the main diffusion direction and/or tractography has been described in several studies [18–23], which show that increasing the number of gradient directions increases fractional anisotropy (FA) and axial diffusivity (AD), while decreasing radial diffusivity (RD) and improving the SNR. Since it involves increased angular resolution, models can be applied beyond the diffusion tensor [24, 25], such as Qball, constrained spherical deconvolution (CSD) and diffusion spectral imaging (DSI) to improve the resolution of fiber crossings [3, 25].
- The influence of the diffusion-sensitizing value (b-value) on the resulting images has been also described, with higher b-values increasing the sensitivity to diffusion, but also increasing noise. The effect of the b-value on anisotropic measures and tractography has been previously studied [21, 26–30].
- Finally, voxel size has a big influence on DWI results. It must be large enough to have an SNR above 3:1 [31], but small enough to minimize the number of voxels containing crossing fiber populations. These two conditions compromise spatial resolution, making it difficult to completely avoid partial volume effects, which vary depending on the type and structure of the tissue [32, 33]. The effect of voxel resolution on DWI results has already been reported [26, 34–37].

In addition to voxel size, it is important to take into account the relationship between its three dimensions, in other words, if the voxel is isometric or anisometric. It has been shown that a bias dependent on fiber bundle orientation is introduced if non-isometric voxels are acquired [38]. However, diffusion studies commonly use anisometric resolution (for example [39–48]) as it solves the limitation of the acquisition time for *in vivo* MRI studies [31]. Losing certain spatial resolution is less problematic when studying the human brain, as it has abundant and large white matter tracts. However, this can be a significant problem for rodents, whose brains are smaller and with less white matter content. Thus, one way to increase the SNR is to increase the voxel size in one direction, raising both the volume and the amount of signal, despite also increasing the fiber population within the voxel. Indeed, a common approach in non-isometric acquisitions is to use multi-slice 2D techniques, which maintain considerable spatial resolution in the image plane, but with a much lower spatial resolution in the slice direction that is necessary to maintain a reasonable SNR. Moreover, the multi-slice 2D techniques are faster to acquire than the 3D techniques.

The influence of anisometric voxels on DWI results may depend on the spatial orientation of the slices with respect to the brain orientation (axial, coronal or sagittal) and on the acquisition orientation (read-, phase- or slice-encoding directions). A quantitative comparison of results obtained with different acquisition techniques and spatial orientations cannot be directly performed as differences in signals arising from anisometric voxels may bias the measurements. These differences are minor when the orientation of the fiber bundle corresponds to the main direction of the anisometric voxel, as the averaging is performed in alignment with the structure. However, if the structure has a different orientation to the main voxel direction, different fiber populations will be averaged. Therefore, the intravoxel orientational dispersion will increase, affecting the quantification of the diffusion anisotropy [31].

This bias will condition all subsequent analyses, affecting both scalar maps calculated from diffusion tensor imaging (DTI) (FA, mean diffusivity (MD), AD and RD) and the streamlines estimated by tractography algorithms. Recently, connectomics has become a common procedure for analyzing brain structure and pathologies [7, 8, 49]. Structural connectomics is based on DWI and tractography streamlines that define the connections between different brain regions. Once the network of brain connections is estimated, graph metrics are used to quantitatively characterize it [7, 8, 50]. Therefore, it is also of great importance to evaluate the influence of DWI acquisition parameters on structural connectomic results.

Here, we present an experimental case study of the effect of anisometric voxels and acquisition orientation on the quantification of diffusion imaging data, namely the estimation of the DTI scalar measures (FA, MD, AD and RD), tractography and analysis of the brain structural connectome.

## Methods

### Animals

Experiments were performed with an adult Wistar male rat, weighing 230 g at the beginning of the study. The rat was housed under controlled temperature (21 ± 1°C) and humidity (55 ± 10%), with a 12-h light/12-h dark cycle (light between 8:00 AM and 8:00 PM). Food and water were available *ad libitum* during all experiments. The rat was sacrificed and transcardially perfused after DWI experiments to dissect the brain for subsequent *ex vivo* experiments not included in this study. Animal work was performed according to local legislation (Decree 214/1997 of July 30^th^ by the ‘Departament d’Agricultura, Ramaderia i Pesca de la Generalitat de Catalunya’) under the approval of the Ethics Committee (CEEA) at the University of Barcelona, and in compliance with European legislation.

### Magnetic resonance imaging

MRI experiments were conducted on a 7.0 Tesla BioSpec 70/30 horizontal animal scanner (Bruker BioSpin, Ettlingen, Germany), equipped with an actively shielded gradients system (400 mT/m, inner diameter of 12 cm). The receiver coil was a 4-channel phased-array surface coil for the rat brain. The animal was placed in a supine position in a plastic holder with a nose cone to administer the anesthetic agent (1.5% isoflurane in a mixture of 30% O_2_ and 70% N_2_O) and was held in place with a tooth bar, ear bars and adhesive tape. Tripilot scans were used to ensure accurate positioning of the head in the magnetic isocenter.

DWI experiments were performed using an EPI diffusion sequence with repetition time TR = 30,000 ms, echo time TE = 39 ms, four segments, b = 1000 s/mm^2^, 81 diffusion directions, five B0 images and one average. TR and TE were selected to be the same in all the acquisitions, giving an acquisition time of 2 hours and 52 minutes for each scan. Using these fixed parameters, 12 acquisitions were undertaken, using three acquisition directions (coronal, axial and sagittal) and four voxel geometries (one isometric and three anisometric).

**Fig 1:**
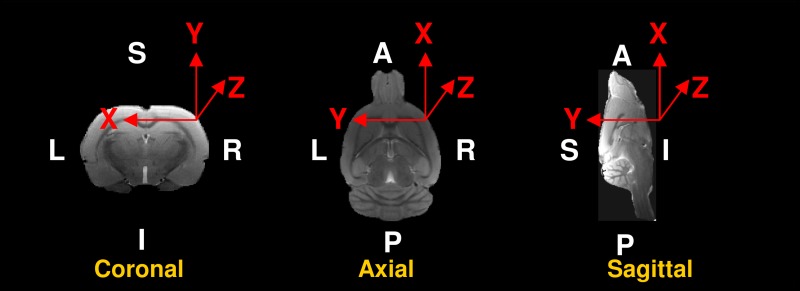
Acquisition orientations. The read (X)-, phase (Y)- and slice (Z)-encoding directions with respect to the anatomical orientation of the rat brain (L, left; R, right; S, superior; I, inferior; P, posterior; and A, anterior) in coronal, axial and sagittal acquisitions.

The three different acquisition directions and their relationship with the rat brain anatomy are shown in Fig 1. The four voxel geometries were acquired in the three possible slice planes, which correspond to the plane containing the read- and phase-encoding directions. The first was the usual coronal rat brain acquisition, with the slices perpendicular to the direction of the main magnetic field, in this case, the read-encoding direction was left to right (LR). The two other acquisitions were the axial (slice-encoding direction superior to inferior (SI)) and sagittal (slice-encoding direction LR) views, both with posterior-to-anterior (PA) read-encoding directions.

**Fig 2:**
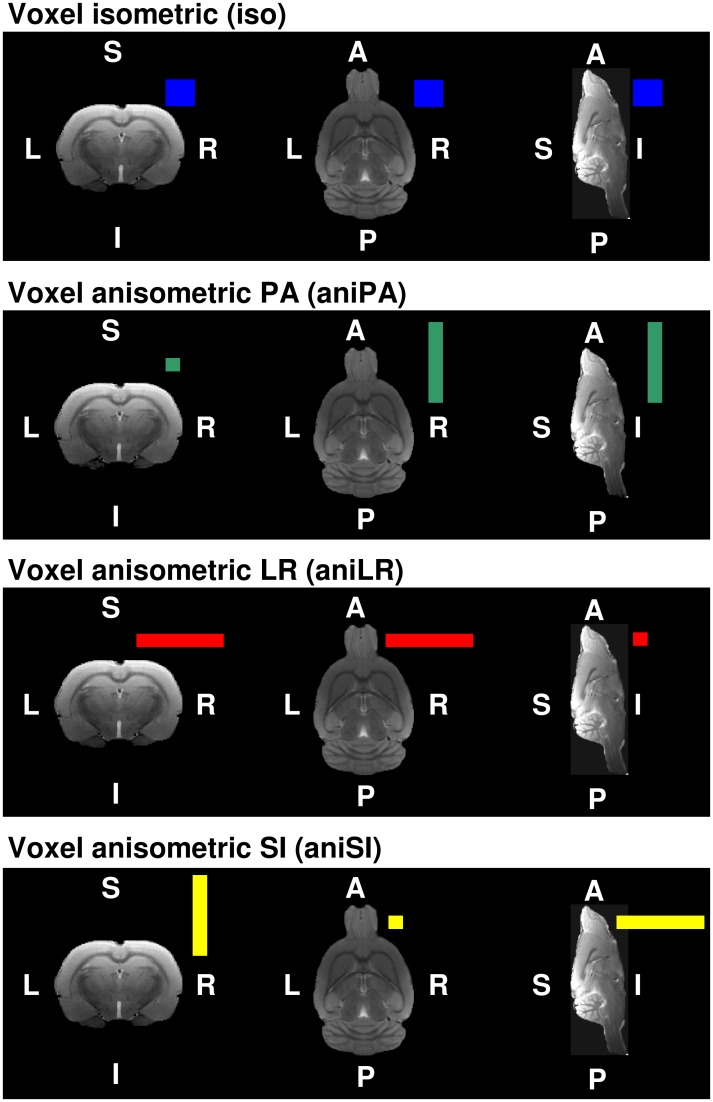
The four voxel geometries used in the acquisitions with respect to the coronal, axial and sagittal views of the rat brain. The isometric voxel is shown in blue, the anisometric voxel in the PA direction in green, the anisometric voxel in the LR direction in red and the anisometric voxel in the SI direction in yellow.

The four voxel geometries are shown in Fig 2. The voxel size was chosen to be similar to that most commonly used in previous works on rat brain diffusion imaging [51, 52]. Isometric and anisometric voxels were also of a similar volume to ensure similar SNRs. The isometric spatial resolution was 0.310 × 0.310 × 0.310 mm^3^, with FOV = 22.32 × 22.32 × 22.32 mm^3^ and matrix size = 72 × 72 × 72 voxels. In the anisometric conditions, voxels had the same size in two of the dimensions (0.155 mm) and a larger size (0.930 mm) in the other. Similar values have been used previously [40–48]. The three anisometric voxels resulted from setting the larger size in the LR, SI and PA rat brain directions, respectively. They were acquired with FOV = 18.6 x 18.6 x 18.6 mm^3^ and matrix size = 120 x 120 x 20 voxels. The size of the anisometric voxels was selected to have proportional dimensions to the isometric voxel, with a similar volume to ensure the same level of signal per voxel in all cases.

Table 1 shows the anisometric voxels expressed with respect to the read/phase/slice-encoding directions and to the anatomical coordinates of the rat brain for the three different acquisition orientations.

**Table 1:** Anisometric voxel orientation.

	Read (aniX)	Phase (aniY)	Slice (aniZ)
**Coronal**	aniLR	aniSI	aniPA
**Axial**	aniPA	aniLR	aniSI
**Sagittal**	aniPA	aniSI	aniLR

Anisometric voxel anatomical orientation correspondence with the read-phase-slice encoding directions of the three different oriented acquisitions.

### Image analysis

#### Image preprocessing

For the sagittal and axial acquisitions, the volume arrays were rearranged to ensure the same orientation and order as those for the coronal acquisitions to simplify posterior image processing and comparison. All these reorientations were performed by just re-sorting the values of the acquired matrix without any interpolation. Before diffusion tensor estimation, reorientation was also applied to the gradient vectors to correctly estimate diffusion directions. In the Bruker system, the gradient vectors are expressed in the read/phase/slice-encoding coordinates system; thus, the application of the gradient vectors also depends on the orientation of the acquisition.

#### Image processing, registration and parcellation

Preprocessing of diffusion images was mainly performed using Dipy [53] and included correcting for eddy currents with FSL [54], denoising using non-local means denoising filtering [55] as implemented in Dipy, and correcting for intensity bias using N4ITK and SimpleITK [56, 57]. Skull stripping was performed based on median Otsu filtering applied to the diffusion-weighted images to extract the brain mask. Diffusion tensor images were estimated, from which scalar maps of FA, MD, AD and RD were calculated. Both whole-brain and regional analyses were carried out for each diffusion scalar map.

For regional analysis, brain parcellation was performed by affine and diffeomorphic registration (ANTS software [58]) of the FA map generated by each acquisition against the average FA map obtained in a previous study [51]. The transformations were applied to a brain atlas to identify the regions in each of the 12 acquisitions. This atlas was an adaptation of the rat brain atlas by Schwarz et al. [59], whose digitized version of that by Paxinos and Watson [60] had 48 composite bilateral structures. It was rescaled to an isometric spatial resolution of 0.31 x 0.31 x 0.31 mm^3^ and manually edited to combine certain structures, resulting in 42 structures per hemisphere. For more details of the atlas construction, please see the Methods section of López et al., 2013 [51].

#### Analysis of DTI scalar maps

For the whole-brain volume (taking into account the voxels obtained from the skull stripping), the mean FA, MD, AD and RD values, standard deviation and histogram were calculated for each acquisition. The histograms had 256 bins ranging from 0 to 1 for FA and from 0 to 0.3·10^−3^ mm^2^/s for the diffusivity parameters. Mean and standard deviation were also calculated for each brain region of the registered FA template. To compare the different acquisition parameters, we used the coronal isometric acquisition as reference, and differences with the other acquisitions were calculated. The distribution of the diffusion parameter in each brain region was represented by a boxplot. Paired Student’s t-tests were performed, comparing the distribution of the values in each region among the acquisitions. Differences were considered significant if p<0.05. Furthermore, a subset of 6 regions from 42 structures were selected to observe specific differences in DWI results for white matter, grey matter and mixed regions with a well-established shape and fiber bundle orientation. The selected regions were: two white matter regions of interest (ROIs), the corpus callosum and internal capsule; one grey matter region, the somatosensory cortex; and three structures containing both white and grey matter, the caudate putamen, anterodorsal hippocampus and dorsolateral thalamus.

#### Connectome construction

A deterministic tractography algorithm based on CSD [61] and the tracking algorithm implemented in Dipy were applied to estimate fiber trajectories. A threshold of FA = 0.15 was used to limit tractography to white matter regions, and all the voxels with FA values higher than 0.2 were considered as seeds for tracking.

The structural brain network was estimated by combining the streamlines resulting from tractography and the regions obtained by brain parcellation. Two regions, A and B, were considered to be connected if there was at least one streamline with endpoints in A and B. Two options were considered to define the weight of a connection: the number of streamlines between two regions (commonly known as fiber number (FN)-weighted connectome) and the mean FA value along the streamlines connecting the regions. In addition, the binary connectome was also considered, where 1 was assigned if two regions were connected and 0 if not.

#### Graph metrics

Standard graph metrics usually calculated in connectomics were assessed for the generated connectomes: degree, weighted strength and global efficiency [50]. The nodal degree is the number of connections of a node. We calculated the network degree as the average nodal degree in the network. Likewise, nodal strength in a weighted connectome was obtained as the sum of the connection weights of a given node. Network strength was the average nodal strength in the whole network. Since we considered two weighted connectomes (FA and FN-weighted), we estimated FA strength and FN strength for each acquisition.

Global efficiency, estimated as the average efficiency for all nodes, reflects integration over the whole network and is related to the inverse of the shortest path length, that is, the distance from a given node to another following the shortest path. Higher values of global efficiency are associated with faster and more efficient communication between nodes. Therefore, while degree and strengths are related to the total number and weight of the connections, global efficiency describes the network organization. Global efficiency was calculated for each of the three connectomes: binary, FA- and FN-weighted.

## Results

### Whole-brain DTI metrics

Mean and standard deviation of the whole-brain FA, MD, AD and RD in the twelve acquisitions are shown in Table 2. Isometric acquisitions yielded similar values regardless of the acquisition orientation. However, when using the anisometric voxel, values depended more on the orientation. When the longest voxel dimension corresponded to the slice-encoding direction (Z) of the acquisition, results were similar to those obtained with isometric voxels. However, when the longest voxel dimension aligned with read (X)- or phase (Y)-encoding directions, there was a decrease in FA and increases in MD, AD and RD. The lowest FA and highest MD, AD and RD values were observed when the longest voxel direction was aligned with the phase-encoding direction (Y). The orientation of the anisometric voxel with respect to the brain anatomy had little effect at this global level.

**Table 2:** Whole-brain DTI parameters.

	FA		MD (10e-3 mm^2/s)		AD (10e-3 mm^2/s)		RD (10e-3 mm^2/s)	
	Mean	SD	Mean	SD	Mean	SD	Mean	SD
**CORONAL**								
Isometric	0.21	0.11	0.73	0.21	0.88	0.24	0.65	0.21
AniPA (Z)	0.20	0.12	0.74	0.20	0.89	0.23	0.67	0.20
AniSI (Y)	0.13	0.07	0.56	0.17	0.63	0.19	0.53	0.16
AniLR (X)	0.17	0.11	0.69	0.22	0.80	0.25	0.64	0.22
**AXIAL**								
Isometric	0.21	0.12	0.73	0.23	0.89	0.26	0.65	0.22
AniSI (Z)	0.21	0.09	0.79	0.17	0.95	0.19	0.70	0.17
AniLR (Y)	0.12	0.06	0.56	0.18	0.63	0.19	0.53	0.17
AniPA (X)	0.15	0.07	0.66	0.16	0.76	0.17	0.61	0.15
**SAGITTAL**								
Isometric	0.22	0.13	0.72	0.23	0.87	0.26	0.65	0.22
AniLR (Z)	0.22	0.14	0.78	0.25	0.94	0.28	0.70	0.24
AniSI (Y)	0.13	0.07	0.55	0.21	0.62	0.23	0.52	0.20
AniPA (X)	0.17	0.12	0.62	0.19	0.72	0.21	0.57	0.18

FA, MD, AD and RD mean and standard deviation in the whole brain volume for the different acquisitions.

**Fig 3:**
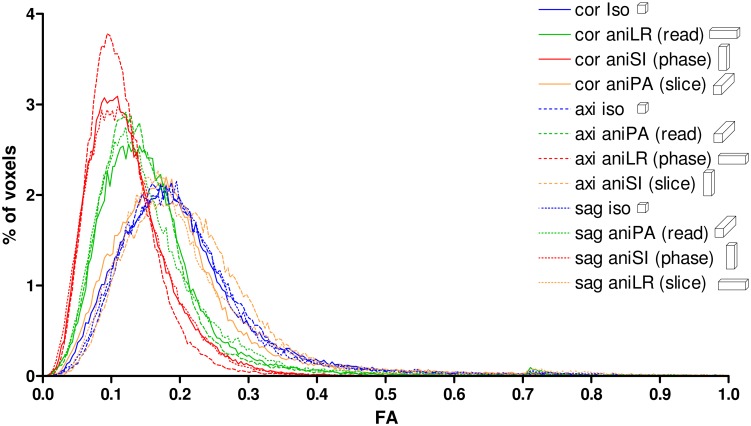
Whole-brain histograms of the FA for the different acquisition orientations with isometric and anisometric voxels.

This behavior was also observed in the whole-brain volume histograms of the FA (Fig 3), where acquisitions with anisometric voxels with the longest axis in the read- or phase-encoding direction showed lower FA values and a narrower distribution compared to the acquisitions with anisometric voxels in the slice-encoding direction, which revealed a distribution more similar to that obtained with the isometric voxels.

### DTI metrics in anatomical brain regions

**Fig 4:**
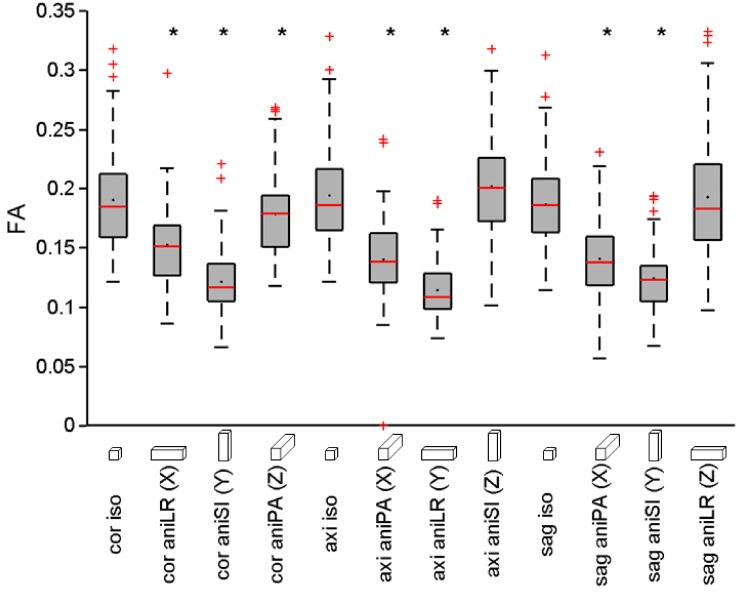
Boxplot of the average FA values of all the regions of the template for the different acquisitions. On each box, the central mark is the median, the black dot the mean, the edges of the box are the 25th and 75th percentiles, the whiskers extend to the most extreme data points and the outliers are plotted individually as red crosses. Asterisks indicate significant difference (p<0.05) between anisometric and isometric acquisitions acquired in the same orientation (coronal, axial or sagittal).

Mean FA, MD, AD and RD values extracted from the template were calculated in each region and compared among the acquisitions. Fig 4 shows the comparison of the distributions of the regional FA mean values for the twelve acquisition configurations. The pattern of differences observed was similar to that obtained with the whole-brain analysis. For the three different orientation acquisitions, there were significant differences (p<0.05) between the mean FA values in the 84 regions of the rat brain using the anisometric voxels oriented in the read- or phase-encoding direction compared to the isometric voxel acquisitions. When the anisometric voxel was oriented in the slice-encoding direction, the difference was only significant for the coronal acquisition. Differences in mean FA values for the different regions were not significant (p>0.05) between coronal, axial and sagittal isometric acquisitions. Similar results were observed for MD, AD and RD in relation to the read-, phase- and slice-encoding orientation (data not shown, see figures in supporting information).

Anisometric acquisitions in the slice-encoding direction gave the closest values to those obtained with isometric acquisitions. Therefore, further analysis was focused only on the three isometric acquisitions and the three anisometric acquisitions in the slice-encoding direction.

Using coronal isometric acquisition as reference, we evaluated the differences in the diffusivity parameters of each region in the five other acquisitions. Table 3 shows the number of regions displaying a significant difference (p<0.05) compared to the coronal isometric acquisition. Diffusivity parameters were significantly different in a high number of regions, indicating that they are affected by both the acquisition orientation and voxel anisometry.

**Table 3:** Number of regions showing significant.

Coronal iso vs	FA	MD	AD	RD
**Sagittal iso**	61	55	52	60
**Axial iso**	51	57	49	52
**Coronal aniPA (Z)**	57	59	53	56
**Sagittal aniLR (Z)**	76	67	66	71
**Axial aniSI (Z)**	67	75	78	73

Number of regions from a total of 84 showing significant differences (p<0.05) in diffusivity parameters among the acquisitions compared to the reference coronal isometric acquisition.

Finally, we evaluated the effect of the orientation of the anisometric voxel with respect to the brain orientation and anatomical structures. The three acquisitions with the anisometric voxel oriented in the slice-encoding direction were performed with the voxel aligned in different directions in relation to the rat brain anatomy: the coronal acquisition was undertaken with the anisometric voxel oriented in the PA direction, the axial with the voxel aligned in the SI direction and the sagittal acquisition was performed with the voxel oriented in the LR direction (see Table 1). Different orientations with respect to the brain anatomy will involve the presence of different mixtures of tissues or structures in each voxel and varying degrees of subsequent partial volume effects that might also have consequences on the diffusivity parameters.

**Fig 5:**
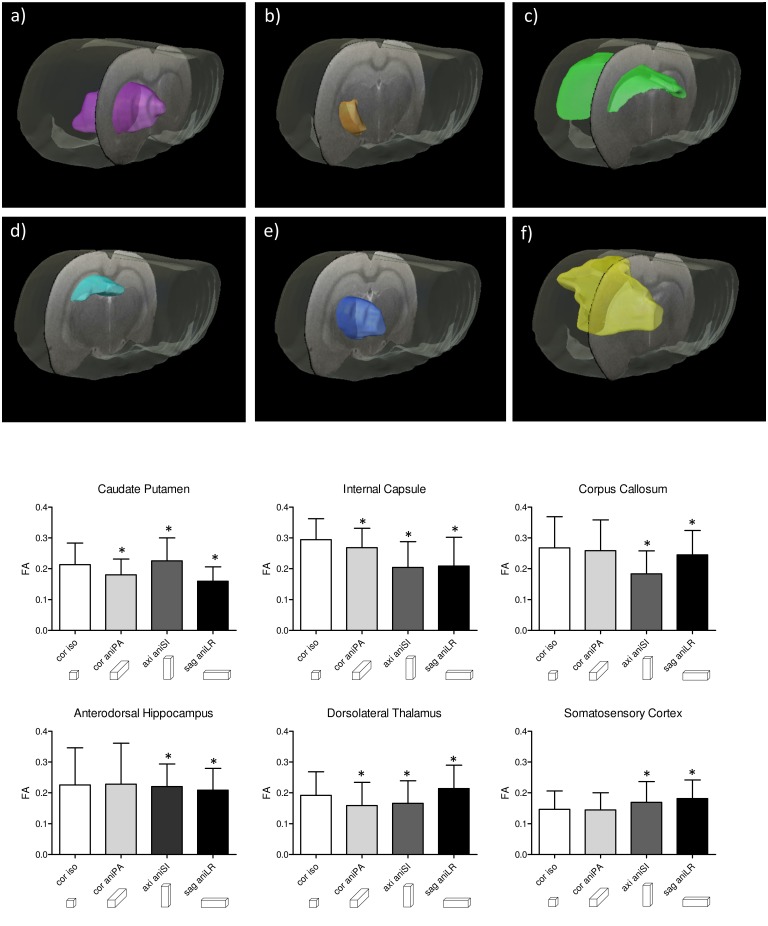
Regions of interest segmented in the right hemisphere and the mean and standard deviation of FA for each region. Regional average FA values in the coronal isometric, coronal aniPA, axial aniSI and sagittal aniLR acquisitions. The (a) caudate putamen, (b) internal capsule, (c) corpus callosum, (d) anterodorsal hippocampus, (e) dorsolateral thalamus and (f) somatosensory cortex. Asterisks show significant differences (p<0.05) in relation to the isometric acquisition.

To evaluate these effects, we selected six regions from the right hemisphere with different tissue types, shapes and orientations. Fig 5 shows the location of these six selected ROIs and the mean and standard deviation values of the FA for each region, with the coronal isometric and the three anisometric acquisitions undertaken in the slice-encoding orientation. The differences between the anisometric and the corresponding isometric acquisitions were significant (p<0.05) in all cases, except for the anisometric PA acquisition in the corpus callosum, somatosensory cortex and anterodorsal hippocampus. In general, the largest difference in the mean FA values was when the anisometric voxel orientation corresponded to the shortest dimension of the region.

### Estimation of fiber orientation

**Fig 6:**
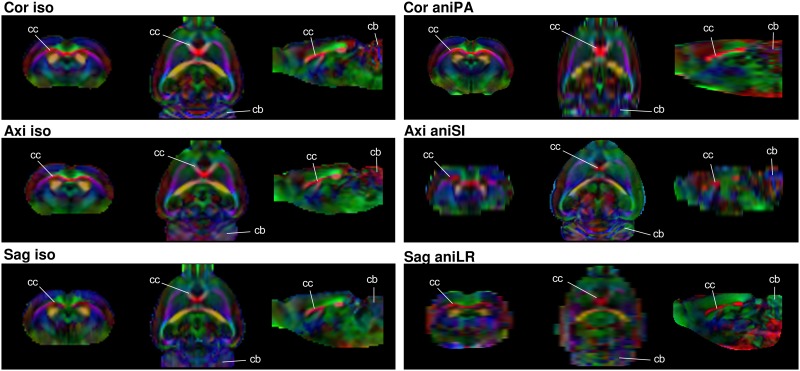
Coronal, axial and sagittal views of the FA color map. Acquisitions in the three orientations using isometric and anisometric voxels in the slice-encoding direction. The white lines indicate the corpus callosum (cc) and cerebellum (cb) locations for anatomic reference.

Fig 6 shows the FA color maps of the six considered acquisitions. The effects of artifacts depended on the orientation, while partial volume effects were more influenced by voxel anisometry.

The FA color maps show the main diffusion direction, estimated as the major eigenvector of the diffusion tensor. Fig 6 shows slices of the isometric coronal, axial and sagittal acquisitions. Differences due to artifacts can be observed, especially in the cerebellum, at one edge of the field of view, where a loss in accuracy was clear in the axial and sagittal acquisitions. The differences can also be observed in the corpus callosum area. In addition, differences in the partial volume effect were observed in the anisometric acquisitions, hampering the identification of some structures in the slices containing the longest voxel dimension.

**Fig 7:**
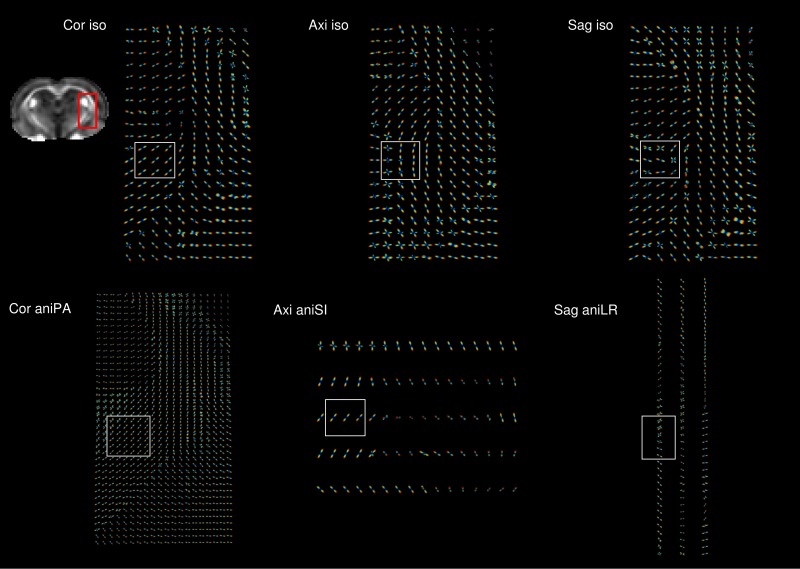
Orientation distribution functions (ODF). Magnification of the same anatomical region (red square) from the coronal view for acquisitions obtained with different orientations and isometric and anisometric voxels in the slice-encoding direction. The white box shows an area where differences between acquisitions can be observed.

Fig 7 displays the glyphs representing the estimated orientation distribution functions (ODF) of the voxels in a region containing white matter structures such as fimbria, the capsula interna and capsula externa. The number of voxels in the region depended on the image resolution. As can be observed from the ODFs estimated from axial and sagittal acquisitions, noise elicited spurious peaks in the ODF that did not correspond to real fiber populations.

Partial volume effects were clearly observed when the resolution was too low. The average diffusion orientations of the ODFs in the area covered by the voxel and therefore, the anisometric configuration, limited the identification of changes in the diffusion orientation along the longest axis.

### Tractography

**Fig 8:**
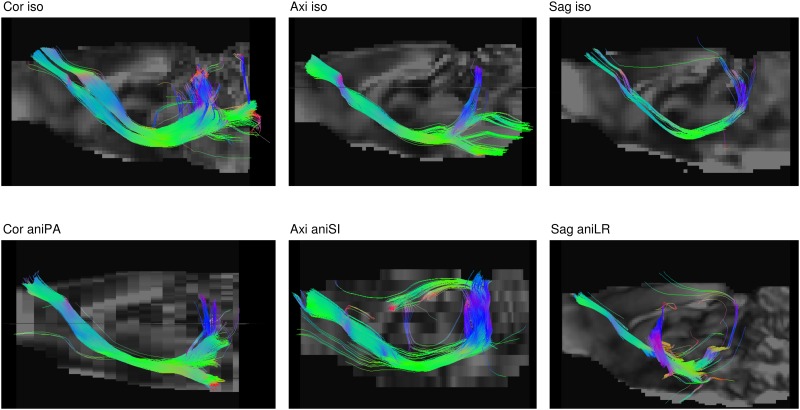
Sagittal view of the corticospinal tract streamlines. Deterministic tractography based on CSD for acquisitions in different orientations and with isometric and anisometric voxels in the slice-encoding direction. Note that in the sagittal anisometric acquisition there are not fibers connecting both regions of interest. In this case, fibers crossing any of the regions have been plotted.

Differences in the estimated ODFs yielded differences in the fiber tracts calculated from them. Fig 8 shows partial streamlines of the corticospinal tract. To identify the tract, the anterior motor prefrontal cortex and capsula interna were manually delineated, and the fibers crossing both regions plotted. Due to artifacts, no tracts satisfied this condition in the sagittal anisometric acquisition. For this reason, fibers crossing each of the areas were plotted, despite not being connected, where this effect can be observed in the sudden change of orientation of the tracts in the center of the image in comparison to the other cases. Table 4 presents the number of streamlines, mean length and FA for the whole brain and the selected tract. Axial and sagittal acquisitions showed more, but shorter streamlines in the whole brain. These acquisitions were noisier and had more artifacts than the coronal acquisition, which might be due to the estimation of spurious directions in some voxels, interrupting the estimation of the continuous fiber path. The anisometric voxel impeded the tracking of the paths, generating more and shorter streamlines.

**Table 4:** Streamline measurements.

**Whole-brain streamlines**		**FA**		**Length (mm)**	
**Acquisition**	**N° streamlines**	**Mean**	**SD**	**Mean**	**SD**
Coronal iso	281270	0.30	0.14	8.13	6.28
Axial iso	290784	0.28	0.12	7.80	5.54
Sagittal iso	310624	0.28	0.13	6.65	4.93
Coronal aniPA (Z)	284416	0.30	0.15	8.43	7.07
Axial aniSI (Z)	397951	0.26	0.08	7.37	5.22
Sagittal aniLR (Z)	365132	0.29	0.15	4.98	3.45
**Corticospinal tract streamlines**		**FA**		**Length (mm)**	
**Acquisition**	**N° streamlines**	**Mean**	**SD**	**Mean**	**SD**
Coronal iso	998	0.25	0.07	18.34	2.53
Axial iso	714	0.27	0.05	17.88	1.13
Sagittal iso	84	0.23	0.06	16.83	3.28
Coronal aniPA (Z)	1474	0.25	0.07	17.57	1.84
Axial aniSI (Z)	1187	0.25	0.06	20.11	3.97
Sagittal aniLR (Z)	-	-	-	-	-

Number of streamlines, mean and standard deviation of FA and the streamline lengths in the whole brain and corticospinal tract. *In the case of sagittal aniLR acquisition, there was no streamlines crossing both anterior motor prefrontal cortex and capsula interna.

### Structural connectome

**Fig 9:**
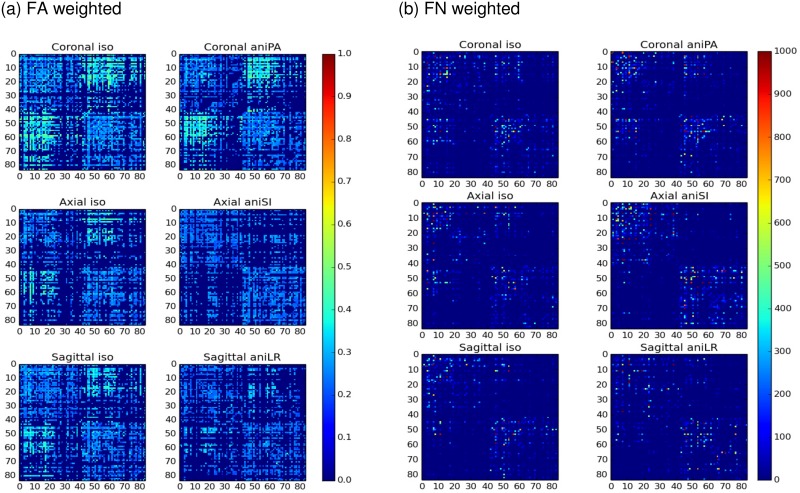
(a) FA-weighted and (b) FN-weighted connectomes. Estimation was undertaken from the acquisitions in the three orientations and with isometric and anisometric voxels in the slice-encoding direction.

Fig 9 shows the FA- and FN-weighted structural connectomes estimated from isometric and anisometric acquisitions in the slice-encoding direction. In S1 Table in supplementary material the correspondence between anatomical regions and labels in the connection matrix is detailed. As a summary, labels from 0–41 corresponds to left hemisphere and 42–83 belongs to right hemisphere, being cortical regions identified with labels from 5 to 17 (left) and from 47 to 59 (right).There were differences among the acquisitions, although the main structure was similar. The differences in FA revealed by the whole-brain analysis were also observed in the FA-weighted connectome, where a higher FA value was observed, especially in the inter-hemispheric cortical connections (connections between areas numbered from 5 to 17 with areas 47 to 59) in the coronal acquisitions. These connections were present with lower FA values in the other isometric acquisitions, but disappeared in the anisometric sagittal and axial acquisitions.

In the FN-weighted connectome, there were differences in the number of streamlines connecting the regions, which directly correlated with the differences observed in tractography. The different distribution of connections showed that a higher number of streamlines did not necessarily mean that more regions were connected.

**Fig 10:**
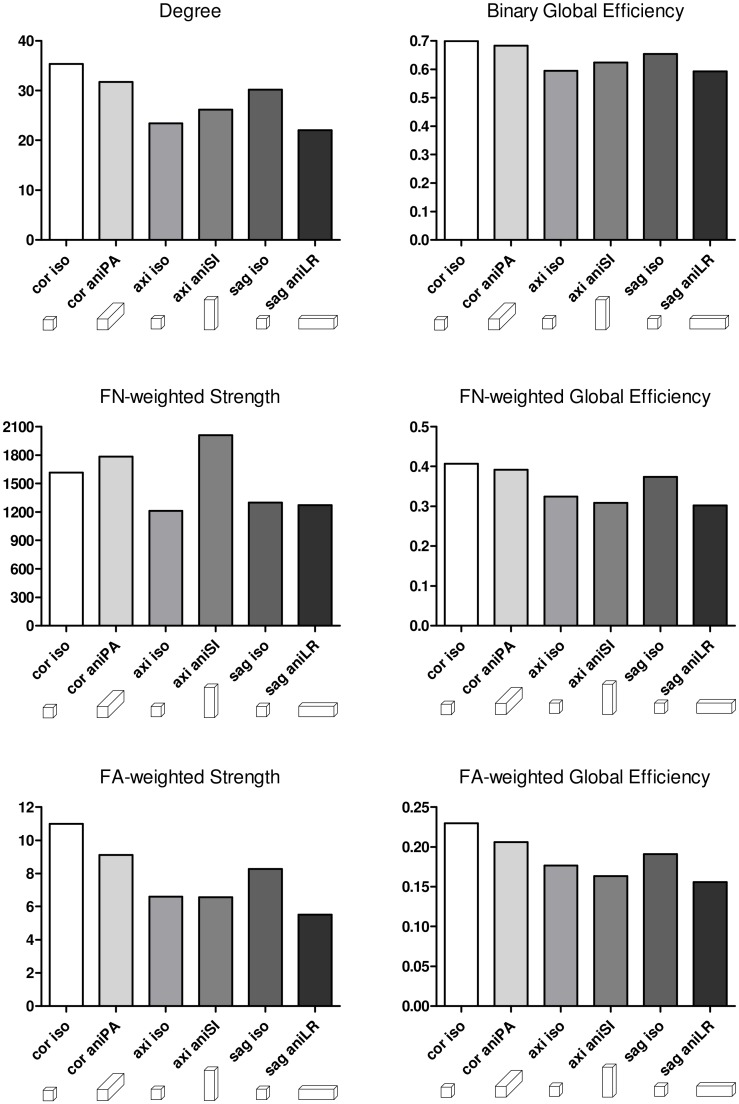
Graph metrics of the binary, FA-weighted and FN-weighted connectomes. Estimation was performed from the three isometric and the three anisometric voxels in the slice-encoding direction acquisitions.

To quantitatively evaluate the connectome, graph metrics were calculated for the three isometric and three anisometric voxels in the slice-encoding direction (Fig 10). Differences among the acquisitions were observed in the degree and strength, measurements of the total amount and/or weight of the connections. The number of interconnected regions is related to the reconstructed streamlines, not only the number but also its length and continuity, since spurious streamlines less likely connect regions. FA- and FN-weighted strength take into account FA and number of streamlines in the connection respectively, and therefore the changes in FA and FN due to acquisition have also an influence in these values, as can be observed comparing them with the number of streamlines and average FA in the whole brain described in Table 4. However, this relation is not straightforward, since connectome is also affected by the quality of the estimated streamlines, as aforementioned. On the other hand, global efficiency metrics, which describe the network organization, are also affected by the acquisition parameters. In this case, the most efficient network was estimated from the coronal isometric acquisition, with similar results obtained with coronal anisometric acquisitions, thus indicating that this metric was more sensitive to changes in the encoding orientation than to voxel anisometry.

## Discussion

Here, we present a case study evaluating the effects of using anisometric voxels, differently oriented in relation to both the rat brain and the magnet, on DWI analysis, particularly standard scalar diffusion parameters and connectomics. Diffusion imaging provides directional information and therefore, the geometry of the acquisition has a direct impact on the results.

Acquisitions were carried out in three possible slice orientations: coronal, axial and sagittal. The slice orientation (Z) not only affects the anatomical view, but also determines the read (X)- and phase (Y)-encoding directions of the acquisition. This has a direct effect on the artifact directions inherent to EPI sequences [2, 3, 14]. EPI images suffer from different image distortions, which include static distortion, due to main field inhomogeneities, and encoding direction-dependent distortion, due to diffusion-weighted gradients. This makes diffusion acquisitions sensitive to artifacts produced by Nyquist ghosting, chemical shift and local susceptibility effects, among others [13, 62]. When considering acquisition orientation, EPI has a limited signal bandwidth in the phase-encoding direction, with local inhomogeneities producing geometric distortion along that direction [63, 64].

Disturbance of field homogeneity is higher in structures where there is a boundary between a high- and a low-susceptibility region, aligned perpendicularly to the B0 field [65]. Furthermore, geometric distortions may be more pronounced when the dominant field gradient is along the phase-encoding direction (due to the limitation in the bandwidth per pixel) [66]. In addition to the orientation of the acquisition, which determines the orientation of the phase-encoding direction, the polarity of the acquisition can also affect the measurement of diffusivity parameters [67].

Another common source of distortion in EPI sequences are eddy currents caused by the fast switching of gradients with large intensities. These eddy current effects are only detectable in the phase-encoding direction [2]. All these artifacts can be reduced during acquisition [68, 69] and with post-processing techniques [64, 70–72], but these corrections could also affect diffusion tensor estimation [73].

The gradient directions used by the MR sequence can also generate very different estimations [74]. We used 81 directions to cover the sphere space uniformly in all the acquisitions, but as the gradient vectors are defined in the read-, phase- and slice-encoding directions, the orientations of these gradients were different with respect to the brain anatomy depending on the acquisition orientation. This could be another source of differences between the acquisitions acquired in axial, coronal and sagittal directions. For instance, the susceptibility distortion, depending on the phase-encoding direction and the location of air boundaries, i.e., the brain orientation, would be different depending on the acquisition. This partly explains the differences observed in the three isometric acquisitions, where voxel geometry was identical, but the acquisition orientation different. Moreover, the inherent structure of the different neuroanatomical regions might be another factor producing the different artifacts observed in each acquisition direction.

Thus, regarding the scalar diffusion parameters (FA, MD, AD and RD), the sagittal and coronal acquisitions showed a high number of regions whose diffusion parameters were significantly different from those obtained in the coronal isometric acquisition, mainly due to the different artifacts associated with each acquisition. However, when analyzing the whole-brain distribution of these values, the three isometric acquisitions showed a similar pattern, indicating a regional effect of the acquisition orientation, which was probably related to the occurrence of artifacts in certain areas. Meanwhile, average global parameters were preserved.

Susceptibility distortion not only affects diffusion tensor estimation and the derived scalar parameters, but also tractography and, therefore, connectivity [75]. The estimation of the diffusion orientation in each voxel is also influenced by the acquisition orientation. Artifacts have a different effect depending on the gradient direction, which might lead to the estimation of spurious directions in the ODFs that do not correspond to the underlying fiber structure. Accordingly, we observed unrealistic secondary peaks in the ODFs estimated from sagittal and axial acquisitions, where the read- or phase-encoding direction was parallel to the main field.

We used a deterministic approach for the tractography algorithm as it is the most widely used for diffusion-based connectomics [49]. The inaccurate estimation of the ODF influences the results of the tractography algorithms, since it hampers the tracking of the path followed by the fibers, leading to disconnections between anatomically-linked regions and the inaccurate assessment of streamlines following unreliable trajectories. This explains the estimation of more, but shorter streamlines in the axial and sagittal acquisitions. The assessment of a higher number of streamlines, however, does not mean better estimation of the fiber structure, as shown when we focused on a specific anatomical tract. In this case, the number of streamlines belonging to the tract was higher when estimated from the coronal acquisition, which produced more reliable tractography results. Moreover, differences in regional FA values among acquisitions can also affect tractography, since the seed points of the algorithm are based on an FA value.

Variations in tractography results directly affect the estimation of brain networks and subsequent connectome analysis, which is based on streamlines to define connections between regions [7, 8]. Thus, we observed higher network metrics in the coronal acquisition, while the lowest values were obtained in the axial acquisition, which had the most artifacts. Differences were clearer in the strength and degree metrics, which measure the number or weight of the connections between regions, but not the network organization, which is measured by global efficiency [50]. Therefore, some of the connections between regions that cannot be inferred from axial or sagittal acquisitions have little influence on the brain network organization, since their impact on global efficiency was not so significant. FA-connectome metrics were more sensitive to the acquisition orientation, probably due to differences in FA.

In addition to the differences associated with the acquisition orientation, we analyzed the effect of using anisometric voxels. Each voxel includes several water compartments endowed with different processes that are mixed up in the data [27]. Therefore, diffusion anisotropy is not only sensitive to the physical and chemical environment, but also to the homogeneity of fiber orientation within the voxel, a more macroscopic property. Thus, diffusion anisotropy can be very sensitive to spatial resolution [76]. In this case, there is an averaging in the longest axis of the voxel. Depending on the orientation of the voxel in relation to the shape of the structure, the incidence of partial volume effects could be significant. This means that the effects of anisometry are closely related to the local structure. In the case of the rat brain, most of the anatomical structures have their longest axis aligned with the PA axis. Hence, if the anisometric voxel is aligned with the long axis of the structure, the resulting average will be more similar to that obtained with an isometric acquisition. However, averaging is performed by mixing fibers from different anatomical regions in LR and SI anisometric acquisitions. Consequently, the partial volume effect is increased, introducing a negative bias in the diffusivity estimation [31]. The varying content of white and grey matter in each voxel also impacts the partial volume effect. Moreover, anatomical regions are identified based on registration against the atlas template, with differences in voxel resolution generating slight differences in the resulting parcellation. All these factors might have contributed to the observed regional differences in scalar diffusion parameters. The analysis of specific regions also showed the varying effect of voxel anisometry depending on the anatomical shape and orientation. Furthermore, brain structures were better identified in the color maps if their main orientation occurred in the highest in-plane resolution. In conclusion, the use of anisometric voxels affected FA, MD, RD and AD values, which tended to be lower in the whole brain.

Partial volume associated with low resolution has a clear effect on ODF estimation. In the longest dimension of the anisometric voxel, resolution was not sufficient to obtain the actual diffusion direction and, therefore, the tractography algorithm could not reliably reconstruct the fiber trajectory. This can be observed in the fiber tract reconstruction plotted in Fig 8. One of the factors that prevented the tracking of the tract connecting the capsula interna and prefrontal cortex in the sagittal acquisition was that voxel anisometry averaged the fiber structure and neighboring structures with different orientations. Moreover, inaccurate ODF estimations led to the assessment of a higher number of streamlines with shorter average lengths, as observed when comparing each anisotropic acquisition to its respective isometric one. Furthermore, differences in FA among the acquisitions also influenced tractography results, since lower FA involves fewer seed points that are considered by the algorithm as points belonging to fiber structures. These changes, together with the differences in defining regions due to varying voxel resolution, might influence the final connectome result. Indeed, higher values for network metrics were obtained in the coronal acquisition for both isometric and anisometric voxels in the PA orientation.

As previously described, anisometric voxels were associated with lower FA values and a higher number of fibers compared to the isometric voxel, which was reflected as lower FA strength and higher FN strength in the anisometric acquisitions. However, when taking into account both the number of connections and the network organization, global efficiency metrics were almost similar for anisometric (in the slice-encoding direction) and isometric acquisitions, with orientation having more of an effect than the anisometric voxel in the slice-encoding direction. Moreover, the higher number of streamlines in the anisometric acquisition did not involve a higher number of connected regions, as can be inferred from the network degree. This is consistent with the tractography results, where we observed a higher number of fibers that were not linked to meaningful anatomical tracts connecting regions. In general, the best organized network (according to the global efficiency metric) was obtained with the isometric coronal acquisition. However, regarding connectome metrics, anisometry in the slice-encoding direction gave more similar results to isometric acquisitions in other directions. This should be taken into account when comparing connectome studies with different voxel size and acquisition parameters, since they have a considerable influence on network metrics.

Voxel size and slice orientation not only affect DWI results, but also those from other techniques involving EPI sequences, such as functional MRI [77]. The aim of this work was to observe the effects of anisometric voxels on different types of diffusion analyses. To quantitatively study these effects, further investigations using larger groups and statistical analysis are required to compare diffusion measurements resulting from acquisitions with different geometries.

## Conclusions

Acquisition direction and voxel geometry significantly influence the results of diffusion-based analysis, as shown by the present study on DT-derived scalar parameters, tractography-based fiber tract estimation and brain network analysis. Hence, it is important to take into account the geometrical aspects of acquisitions when comparing diffusion results obtained from different equipment or studies using the same magnet, but different acquisition conditions.

## Supporting Information

S1 Fig Boxplot of the average MD (mm^2^/s) values of all the regions of the template for the different acquisitions. On each box, the central mark is the median, the black dot the mean, the edges of the box are the 25th and 75th percentiles, the whiskers extend to the most extreme data points and the outliers are plotted individually as red crosses.
(TIF)

S2 Fig Boxplot of the average AD (mm^2^/s) values of all the regions of the template for the different acquisitions. On each box, the central mark is the median, the black dot the mean, the edges of the box are the 25th and 75th percentiles, the whiskers extend to the most extreme data points and the outliers are plotted individually as red crosses.
(TIF)

S3 Fig Boxplot of the average RD (mm^2^/s) values of all the regions of the template for the different acquisitions. On each box, the central mark is the median, the black dot the mean, the edges of the box are the 25th and 75th percentiles, the whiskers extend to the most extreme data points and the outliers are plotted individually as red crosses.
(TIF)

S1 Table List of regions. ID number for each region of the atlas used in the connectomics.
(PDF)
