# Supplementary figures and images for: Effects of Orientation and Anisometry of Magnetic Resonance Imaging Acquisitions on Diffusion Tensor Imaging and Structural Connectomes
Source: PLoS One. 2017 Jan 24;12(1):e0170703. doi: 10.1371/journal.pone.0170703 (PMC5261617; doi:10.1371/journal.pone.0170703)

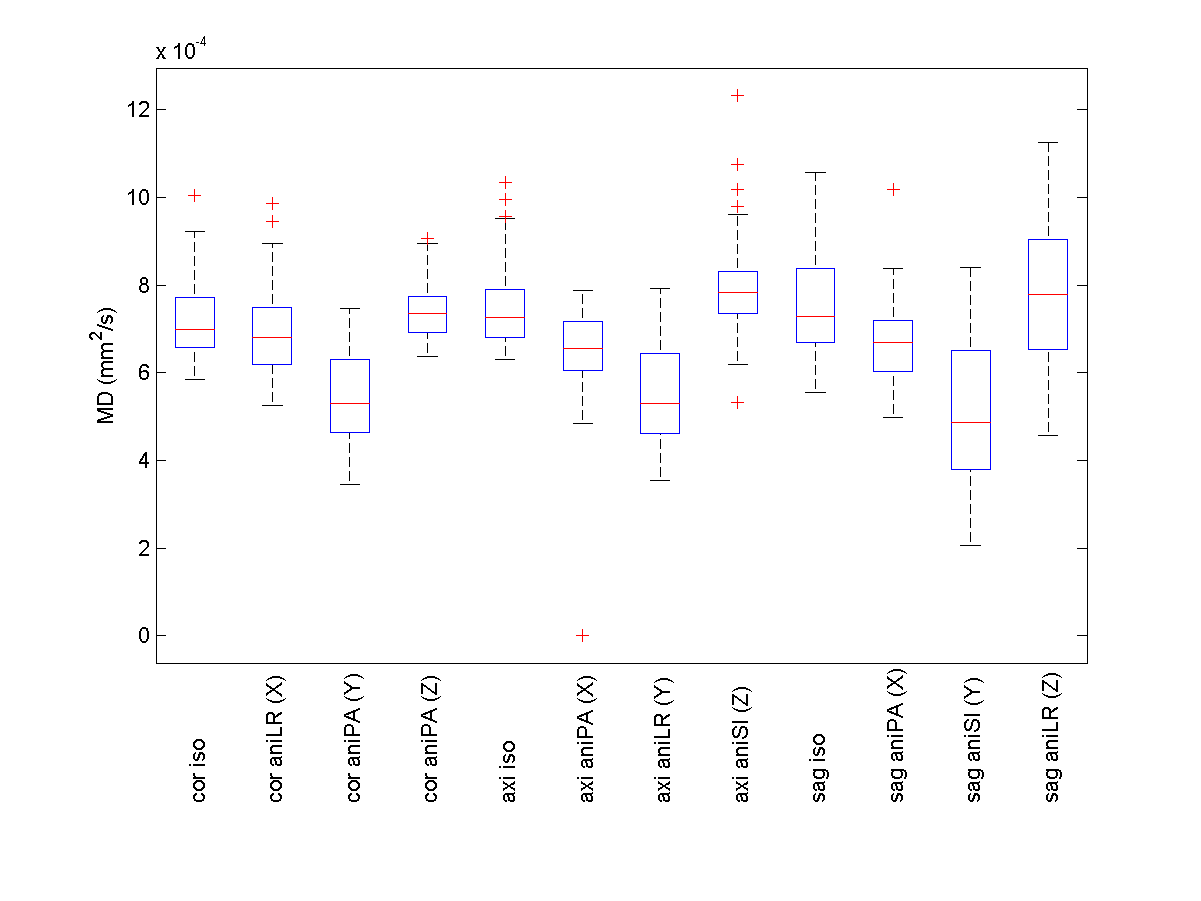

Supplement: S1 Fig — On each box, the central mark is the median, the black dot the mean, the edges of the box are the 25th and 75th percentiles, the whiskers extend to the most extreme data points and the outliers are plotted individually as red crosses. (TIF) [file pone.0170703.s001.tif]

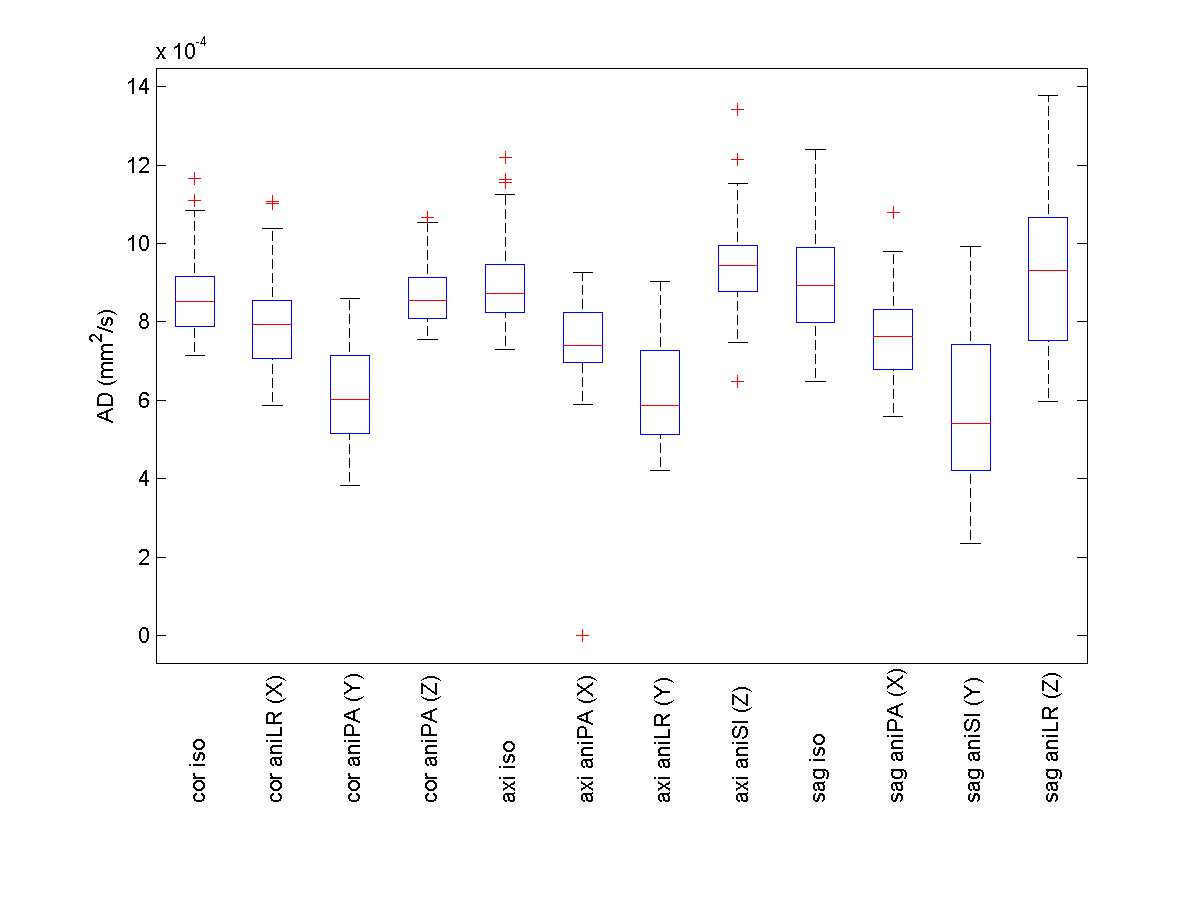

Supplement: S2 Fig — On each box, the central mark is the median, the black dot the mean, the edges of the box are the 25th and 75th percentiles, the whiskers extend to the most extreme data points and the outliers are plotted individually as red crosses. (TIF) [file pone.0170703.s002.tif]

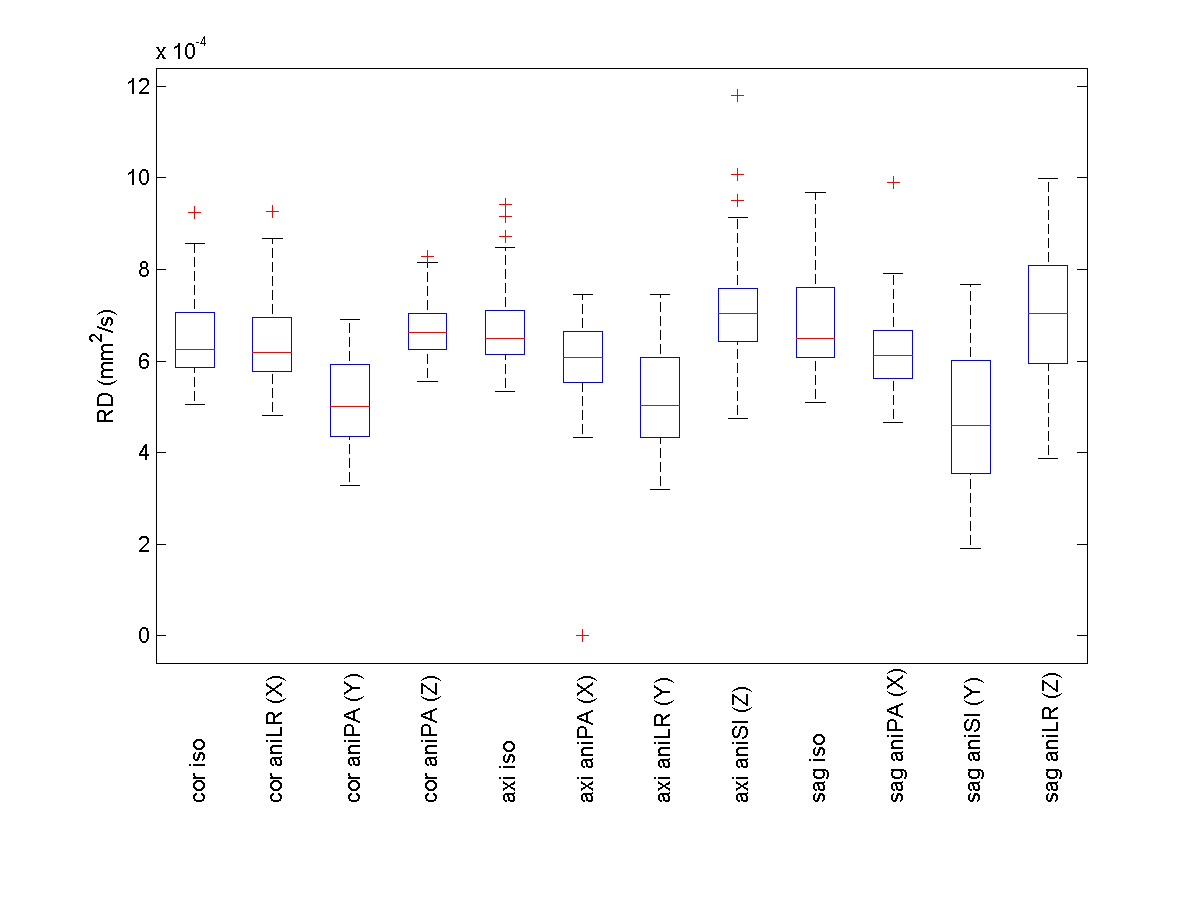

Supplement: S3 Fig — On each box, the central mark is the median, the black dot the mean, the edges of the box are the 25th and 75th percentiles, the whiskers extend to the most extreme data points and the outliers are plotted individually as red crosses. (TIF) [file pone.0170703.s003.tif]
