# Supplementary material for: Effects of Orientation and Anisometry of Magnetic Resonance Imaging Acquisitions on Diffusion Tensor Imaging and Structural Connectomes
Source: PLoS One. 2017 Jan 24;12(1):e0170703. doi: 10.1371/journal.pone.0170703 (PMC5261617; doi:10.1371/journal.pone.0170703)
Supplement: S1 Table — ID number for each region of the atlas used in the connectomics. (PDF) [file pone.0170703.s004.pdf]

LEFT	REGION	RIGHT
0	accumbens	42
1	amygdala	43
2	bed nucleus stria terminalis	44
3	caudate putamen	45
4	corpus callosum	46
5	auditory cortex	47
6	cingulate cortex	48
7	entorhinal cortex	49
8	frontal association cortex	50
9	insular cortex	51
10	medial prefrontal cortex	52
11	motor cortex	53
12	orbitofrontal cortex	54
13	parietal association cortex	55
14	retrosplenial cortex	56
15	somatosensory cortex	57
16	temporal association cortex	58
17	visual cortex	59
18	globus pallidus	60
19	hippocampus antero dorsal	61
20	hippocampus posterior	62
21	hippocampus postero dorsal	63
22	hippocampus ventral	64
23	hypothalamus	65
24	internal capsule	66
25	medial geniculate	67
26	mesencephalic region	68
27	olfactory tubercle	69
28	periaqueductal gray	70
29	pons	71
30	raphe	72
31	septum	73
32	substantia innominata	74
33	substantia nigra	75
34	superior colliculus	76
35	thalamus dorsolateral	77
36	thalamus midline dorsal	78
37	thalamus ventromedial	79
38	ventral pallidum	80
39	ventral tegmental area	81
40	anterior commissure	82
41	fimbria	83

S1 Table. List of regions. ID number for each region of the atlas used in the connectomics.
